# Impact of HER2‐low expression on the efficacy of endocrine therapy with or without CDK4/6 inhibitor in HR‐positive/HER2‐negative metastatic breast cancer: A prospective study

**DOI:** 10.1111/1759-7714.15282

**Published:** 2024-03-13

**Authors:** Yun Wu, Hongnan Mo, Hangcheng Xu, Yan Wang, Jiayu Wang, Fei Ma, Binghe Xu

**Affiliations:** ^1^ Department of Medical Oncology, National Cancer Center/National Clinical Research Center for Cancer/Cancer Hospital Chinese Academy of Medical Sciences and Peking Union Medical College Beijing China

**Keywords:** CDK4/6 inhibitors, endocrine therapy, HER2‐low, metastatic breast cancer, prospective analysis

## Abstract

**Background:**

CDK4/6 inhibitors in combination with traditional endocrine therapy (ET) have become the recommended first‐line therapy for HR‐positive/HER2‐negative metastatic breast cancer (MBC). The aim of this prospective study was to evaluate the relationship between HER2‐low expression and clinical outcomes in HR‐positive/HER2‐negative MBC patients receiving ET with or without CDK4/6 inhibitors.

**Methods:**

Between April 2016 and November 2019, 233 women with HR‐positive/HER2‐negative MBC who received ET with or without CDK4/6 inhibitors were enrolled into the study. The primary endpoint was progression‐free survival (PFS). Statistical analysis included descriptive statistics, Kaplan–Meier curves, and Cox proportional hazards models.

**Results:**

HER2‐low and HER2‐zero subgroups in the CDK4/6 inhibitor plus ET cohort showed no significant difference in the median PFS (10.9 vs. 8.0 months; hazard ratio: 0.92; 95% confidence interval [CI]: 0.64–1. 30; *p* = 0.65), while HER2‐low subgroup showed a significantly shorter median PFS compared to the HER2‐zero subgroup in the ET alone cohort (5.6 vs. 17.0 months; hazard ratio: 2.82; 95% CI: 1.34–5.93; *p* = 0.0044). Moreover, the objective response rate was significantly lower in the HER2‐low subgroup than the HER2‐zero subgroup in the ET alone cohort (10.5% vs. 40.0%, *p* = 0.047). Lastly, no significant difference was observed in the overall survival between the HER2‐low and HER2‐zero subgroups in both cohorts.

**Conclusion:**

This study suggested that HER2‐low expression may predict the efficacy of ET but not that of CDK4/6 inhibitor plus ET in HR‐positive/HER2‐negative MBC patients. The results of this study highlight the importance of integrating HER2 status in tailoring personalized treatment strategies for HR‐positive MBC.

## Graphical abstract

- HER2‐low tumors demonstrated a less favorable prognosis compared to HER2‐zero tumors when treated with endocrine therapy alone. However, no survival difference observed in HER2‐low and HER2‐zero patients with HR‐positive/HER2‐negative metastatic breast cancer treated with CDK4/6 inhibitors plus endocrine therapy.
- HER2‐low expression might serve as a potential biomarker for assessing the efficacy of endocrine therapy in metastatic breast cancer.

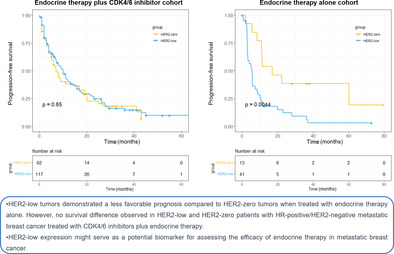

## INTRODUCTION

Breast cancer (BC) is the most common malignancy among women, with over 2 260 000 newly diagnosed cases and nearly 685 000 deaths reported worldwide.^1^ BC can be classified into four distinctive molecular subtypes based on the expression of human epidermal growth factor receptor 2 (HER2) and hormone receptor (HR), providing a solid reference for precise treatment and prognosis estimation.^2^

HER2‐low breast cancer defined as immunohistochemistry (IHC) 1+ or IHC 2+ with a negative in situ hybridization (ISH) assay, which accounts for 45%–55% of breast cancer patients, is less likely to benefit from conventional HER2‐targeting drugs. Recently, the new generation of HER2‐directed antibody–drug conjugates, particularly trastuzumab deruxtecan, have shown substantial efficacy in HER2‐low BC, suggesting a possibility of improving the clinical interpretation of HER2 status from the current binary to a ternary pattern.^3^ HER2‐low BC constitutes a molecularly diverse and clinically heterogeneous group, comprising the majority of HR‐positive tumors. However, it is still unclear whether HER2‐low BC can be considered as a distinct biological subtype compared to HER2‐zero BC.

Therapeutic strategies for HER2‐low metastatic BC (MBC) are primarily determined by the HR status. Cyclin‐dependent kinase 4/6 (CDK4/6) inhibitors combined with traditional endocrine therapy (ET) has become the recommended first‐line therapy for patients with HR‐positive MBC and is widely used in clinical practice.^4^ However, the generation of CDK4/6 inhibitor resistance and the identification of potential biomarkers to predict ET efficacy are a few concerns associated with this treatment. Crosstalk between ER and HER2 signals has been predicted to contribute to ET resistance. Studies have suggested that HER2‐low tumor cells could express approximately 40 000 to 100 000 HER2 per cell at the single‐cell level, indicating a potential effect on CDK4/6 inhibitor efficacy.^5^ Recently, a retrospective analysis of 106 HR‐positive HER2‐negative MBC patients treated with ET plus CDK4/6 inhibitors found an inferior progression‐free survival (PFS) in the HER2‐low group compared to the HER2‐zero group.^6^ In contrast, Carlino et al.^7^ investigated 165 HR‐positive MBC patients who received ET plus palbociclib and reported similar clinical outcomes in the HER2‐low and HER2‐zero groups.

To date, given the potentially insufficient sample volume and retrospective study design, the clinical impact of the HER2 low expression on the response to endocrine therapy with or without CDK4/6 inhibitors in patients with MBC remains controversial. This prospective study aimed to evaluate the relationship between HER2 low expression and clinical outcomes in HR‐positive/HER2‐negative MBC patients receiving ET combined with or without CDK4/6 inhibitors, and hopefully to help the clinician select personalized treatment strategies for HR‐positive HER2‐low BC patients.

## METHODS

### Study design and participants

For the ET alone cohort, data were obtained from the FRIEND prospective phase 2 clinical trial (NCT02646735), conducted at the subcenter of the Cancer Hospital, Chinese Academy of Medical Sciences (CHCAMS; Beijing, China) between April 2016 and November 2019. This study enrolled postmenopausal women with estrogen receptor (ER)‐positive/HER2‐negative MBC who had not received prior systemic treatment. These patients were randomly assigned to receive either exemestane or fulvestrant in a 1:1 ratio. The detailed study design and eligibility criteria for this study have been published previously.^8^ For the CDK4/6 inhibitor plus ET cohort, eligible patients were recruited from those enrolled in the CHCAMS between May 2016 and November 2019. Only patients who had confirmed HR‐positive/HER2‐negative MBC and were scheduled to receive palbociclib in combination with ET were enrolled in this study. Patients with incomplete pathological data or insufficient follow‐up information were excluded from this study.

Baseline characteristics, including age, menopausal status, Eastern Cooperative Oncology Group (ECOG) performance status, histological type, HR and HER2 status, treatment‐free intervals (TFIs), previous adjuvant treatment, surgery prior to metastasis, disease status, ET agents, and previous endocrine therapies for metastasis, were collected in this study. The patients in the two cohorts were stratified into two subgroups based on HER2 expression (HER2‐low vs. HER2‐zero).

This study was approved by the Ethics Committee of the CHCAMS (19/331–2115). All patients provided written informed consent. The study conformed to the Helsinki Declaration and followed the guidelines of Good Clinical Practice.

### Study endpoints and assessment

The primary endpoint in this study was PFS, which is defined as the time from the initiation of ET to disease progression or death due to any cause. The secondary endpoints in this study included overall survival (OS), objective response rate (ORR), and disease control rate (DCR). OS is defined as the time from the initiation of treatment to death due to any cause. ORR is defined as the proportion of patients achieving a complete response (CR) or partial response, while DCR is defined as the proportion of patients achieving CR, partial response, and stable disease. HR‐positive was characterized as positive for either ER and/or progesterone receptor (PR), with a threshold of ≥1% staining positivity. Within HER2‐negative tumors, HER2‐low status was identified by an IHC score of 1+ or 2+ and by negative ISH, while HER2‐zero status was identified by an IHC score of 0.

### Statistical analysis

Descriptive statistics were used to summarize the baseline characteristics of the study population. Kaplan–Meier curves were used to estimate the PFS and OS. Hazard ratios and their 95% confidence intervals (CIs) were calculated using Cox proportional hazards models. The PFS and OS were analyzed according to the HER2 status (HER2‐low vs. HER2‐zero) to evaluate the impact of HER2‐low status on treatment efficacy. To control for potential confounding and heterogeneity, adjusted hazard ratios were estimated within each treatment cohort using two different multivariable Cox models. In addition, response rates, such as ORR and DCR, were compared between the HER2‐low and HER2‐zero patients in the two cohorts by Pearson's Chi‐squared test or Fisher's exact test. All statistical analyses were performed using SPSS version 26.0 (SPSS Inc.) and the R software version 4.1.1. A two‐sided *p*‐value of <0.05 was considered statistically significant.

## RESULTS

### Characteristics of study population

Among the 233 patients enrolled in this study, the CDK4/6 inhibitor plus ET cohort (Table 1) consisted of 117 (65.4%) HER2‐low patients (61 [52.1%] HER2 IHC 1+ and 56 [47.9%] HER2 IHC 2+/ISH‐negative) and 62 (34.6%) HER2‐zero patients, while the ET alone cohort (Table 2) consisted of 41 (75.9%) HER2‐low patients (18 [43.9%] HER2 IHC 1+ and 23 [56.1%] HER2 IHC 2+/ISH‐negative) and 13 (24.1%) HER2‐zero patients. The baseline characteristics within the CDK4/6 inhibitor plus ET cohort were generally balanced across the HER2‐low and HER2‐zero subgroups, except for the ER/PR status (*p* = 0.004). In the ET alone cohort, the baseline characteristics were generally comparable between the two HER2 subgroups except TFIs after the end of adjuvant (*p* = 0.033).

**TABLE 1:** Baseline characteristics of the patients in the CDK4/6 inhibitor plus ET cohort based on the HER2 status.

	All patients (*N* = 179)	HER2‐low (*N* = 117)	HER2‐zero (*N* = 62)	*p*‐value^a^
Age				0.373
Mean (SD)	46.46 (11.39)	46.66 (11.74)	46.10 (10.79)	
Median (Range)	46 (19–83)	46 (19–83)	45 (29–72)	
Age group				
<65	163 (91.06)	107 (91.45)	56 (90.32)	0.801
≥65	16 (8.94)	10 (8.55)	6 (9.68)	
Menopausal status				0.662
Postmenopausal	74 (41.34)	47 (40.17)	27 (43.55)	
Premenopausal	105 (58.66)	70 (59.83)	35 (56.45)	
Histological type				0.141
IDC	157 (87.71)	103 (88.03)	54 (87.10)	
ILC	9 (5.03)	8 (6.84)	1 (1.61)	
Others	13 (7.26)	6 (5.13)	7 (11.29)	
ER/PR status				0.004**
ER+/PR+	15 (87.15)	108 (92.31)	48 (77.42)	
ER+/PR−	20 (11.17)	9 (7.68)	11 (17.74)	
ER−/PR−	3 (1.68)	0	3 (4.84)	
HER2 status				–
IHC 0	62 (34.64)	–	62 (100.00)	
IHC 1+	61 (34.08)	61 (52.14)	–	
IHC 2+/ISH‐negative	56 (31.28)	56 (47.86)	–	
ECOG score				0.465
0	47 (27.49)	30 (27.03)	17 (28.33)	
1	116 (67.84)	74 (66.67)	42 (70.00)	
≥2	8 (4.68)	7 (6.31)	1 (1.67)	
Missing	8	6	2	
Site of metastasis				0.911
Bone or soft tissue	50 (27.93)	33 (28.21)	17 (27.42)	
Visceral metastasis	129 (72.07)	84 (71.79)	45 (72.58)	
Previous adjuvant treatments				
Chemotherapy	134 (74.86)	85 (72.65)	49 (79.03)	0.349
ET	119 (66.48)	74 (63.25)	45 (72.58)	0.208
Surgery prior to metastasis				0.306
Yes	164 (91.62)	109 (93.16)	55 (88.71)	
No	15 (8.38)	8 (6.84)	7 (11.29)	
Disease status				0.985
Initial diagnosis stage IV	29 (16.20)	19 (16.24)	10 (16.13)	
Recurrence and metastasis	150 (83.80)	98 (83.76)	52 (83.87)	
Combination ET agents				0.736
AI	80 (44.69)	50 (42.74)	30 (48.39)	
Fulvestrant	88 (49.16)	60 (51.28)	28 (45.16)	
Tamoxifen/torimifene	11 (6.15)	7 (5.98)	4 (6.45)	
Previous ETs for metastasis				0.336
0	78 (43.58)	52 (44.44)	26 (41.93)	
1	41 (22.90)	23 (19.66)	18 (29.03)	
≥2	60 (33.52)	42 (35.90)	18 (29.03)	

Abbreviations: AI, aromatase inhibitors; CDK4/6, cyclin‐dependent kinase 4/6; ECOG, Eastern Cooperative Oncology Group; ER, estrogen receptor; ET, endocrine therapy; SD, standard deviation; HER2, human epidermal growth factor receptor 2; IDC, invasive ductal carcinoma; ILC, invasive lobular carcinoma; PR, progesterone receptor.

** *p <* 0.01.

^a^ Wilcoxon rank sum test; Pearson's chi‐squared test; Fisher's exact test.

**TABLE 2:** Baseline characteristics of the patients in the ET alone cohort based on the HER2 status.

	All patients (*N* = 54)	HER2‐low patients (*N* = 41)	HER2‐zero patients (*N* = 13)	*P*‐value^a^
Age				0.649
Mean (SD)	62.81 (5.26)	62.83 (5.45)	62.77 (4.80)	
Median (range)	62 (54–76)	62 (54–76)	63 (55–71)	
Age group				0.653
<65	36 (66.67)	28 (68.29)	8 (61.54)	
≥65	18 (33.33)	13 (31.71)	5 (38.46)	
Histological type				>0.999
IDC	51 (94.45)	38 (92.68)	13 (100.00)	
ILC	1 (1.85)	1 (2.44)	0	
Others	2 (3.70)	2 (4.88)	0	
ER/PR status				0.653
ER+/PR+	48 (88.89)	36 (87.80)	12 (92.31)	
ER+/PR−	6 (11.11)	5 (12.20)	1 (7.69)	
ER−/PR−				
HER2 status				–
IHC 0	13 (24.08)	–	13 (100.00)	
IHC 1+	18 (33.33)	18 (43.90)	–	
IHC 2+/ISH‐negative	23 (42.59)	23 (56.10)	–	
ECOG score				0.821
0	34 (62.96)	26 (63.41)	8 (61.54)	
1	19 (35.19)	14 (34.15)	5 (38.46)	
≥2	0	0	0	
Missing	1 (1.85)	1 (2.44)	0	
Site of metastasis				0.736
Bone or soft tissue	20 (37.04)	15 (31.71)	5 (38.46)	
Visceral metastasis	34 (62.96)	26 (63.41)	8 (61.54)	
Previous adjuvant treatments				
Chemotherapy	51 (94.44)	40 (97.56)	11 (84.62)	>0.999
Endocrine therapy	52 (96.30)	40 (97.56)	12 (92.31)	>0.999
ET agents				0.637
AI	26 (48.15)	19 (46.34)	7 (53.85)	
Fulvestrant	28 (51.85)	22 (53.66)	6 (46.15)	
TFI post‐adjuvant ET				0.033*
≥12 months	18 (33.33)	10 (24.39)	8 (61.54)	
<12 months	36 (66.67)	31 (75.61)	5 (38.46)	

Abbreviations: AI, aromatase inhibitors; ECOG, Eastern Cooperative Oncology Group; ER, estrogen receptor; ET, endocrine therapy; SD, standard deviation; HER2, human epidermal growth factor receptor 2; IDC, invasive ductal carcinoma; ILC, invasive lobular carcinoma; PR, progesterone receptor; TFI, treatment‐free interval.

* *p <* 0.05.

^a^ Wilcoxon rank sum test; Pearson's chi‐squared test; Fisher's exact test.

### Clinical outcomes

**FIGURE 1:**
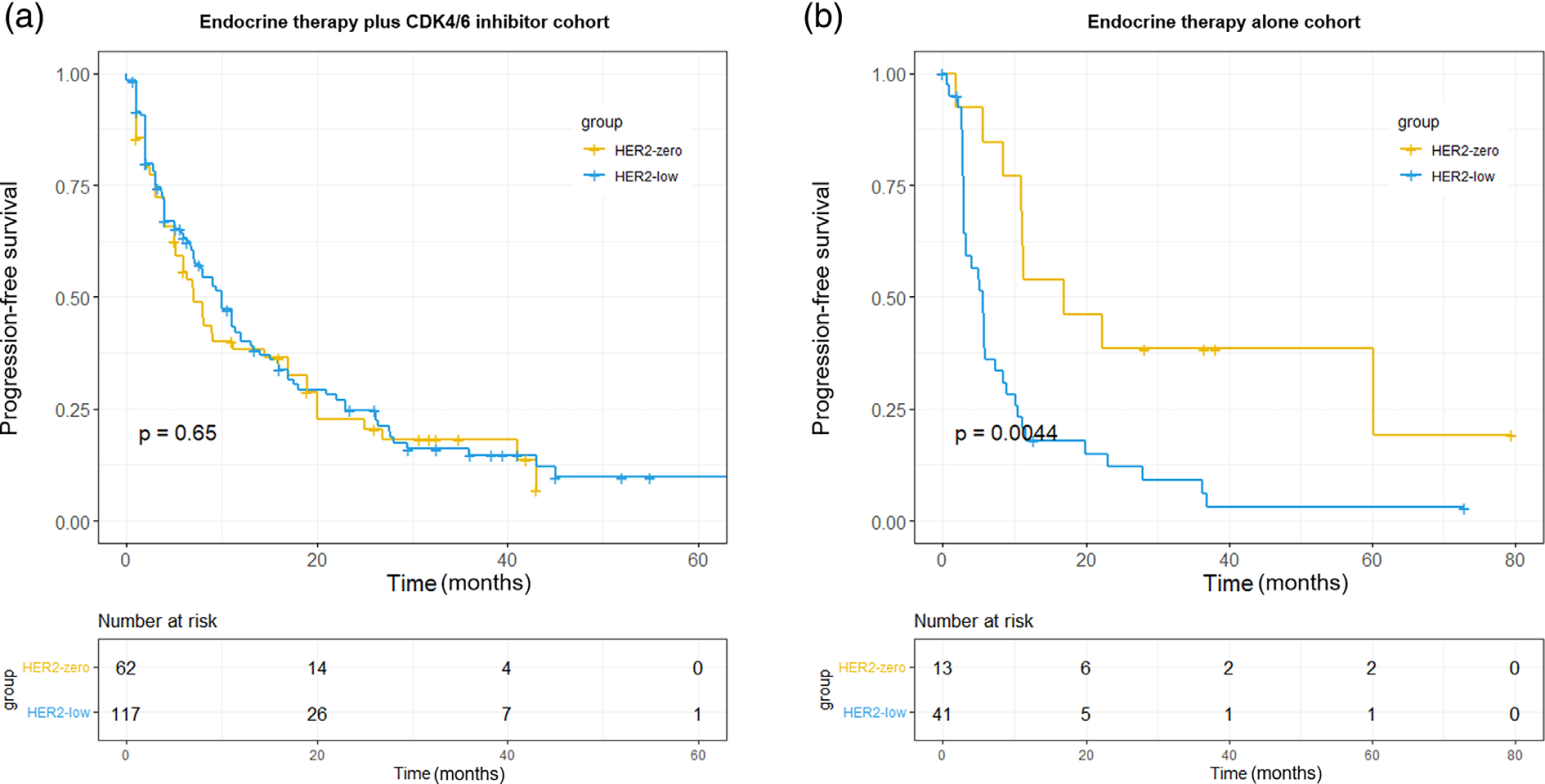
Kaplan–Meier estimates of progression‐free survival of patients in the CDK4/6 inhibitor plus ET cohort (a) and ET alone cohort (b) stratified by the HER2 status. CDK4/6, cyclin‐dependent kinase 4/6; ET, endocrine therapy; HER2, human epidermal growth factor receptor 2.

**FIGURE 2:**
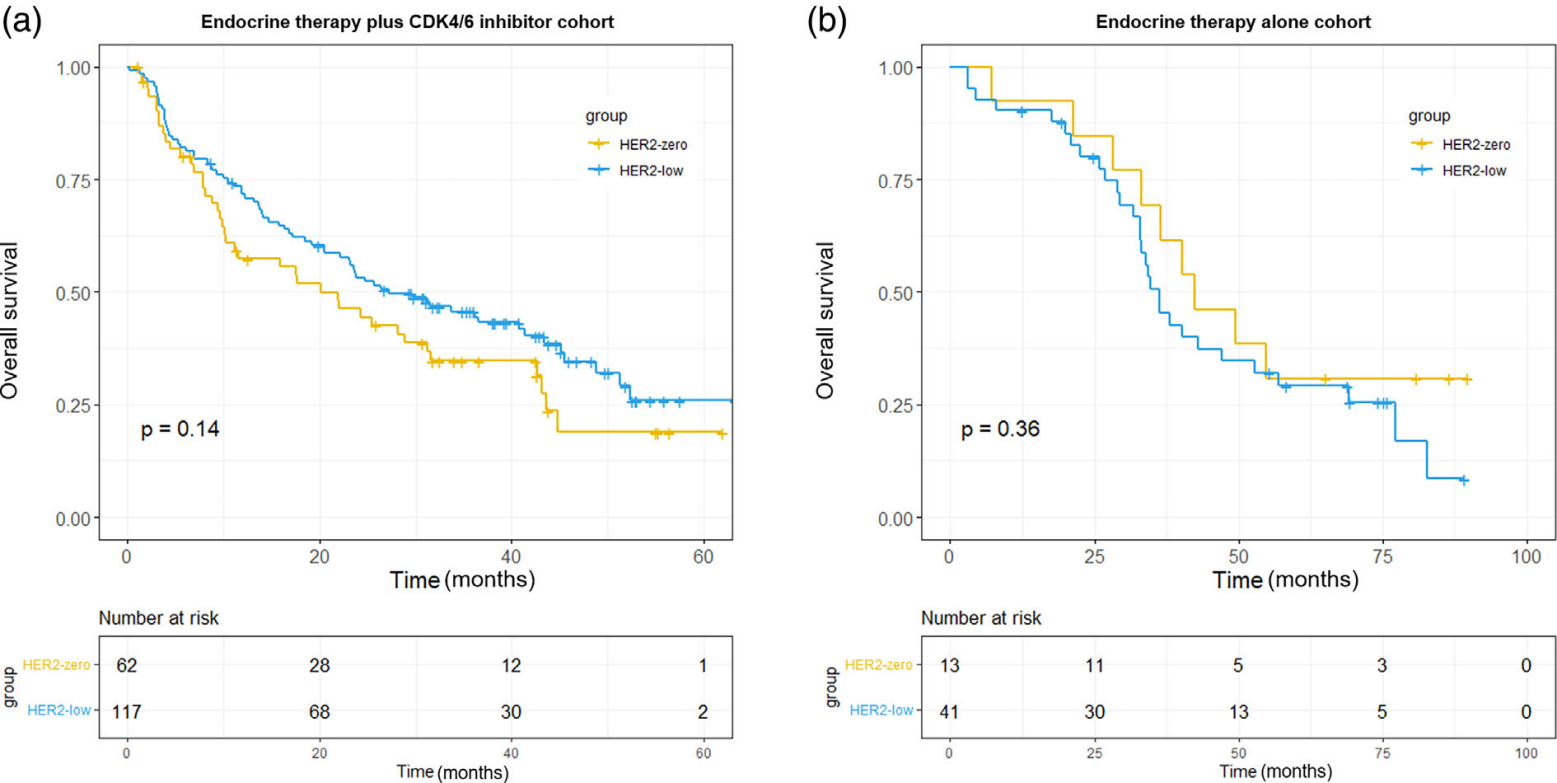
Kaplan–Meier estimates of overall survival of patients in the CDK4/6 inhibitor plus ET cohort (a) and ET alone cohort (b) stratified by the HER2 status. CDK4/6, cyclin‐dependent kinase 4/6; ET, endocrine therapy; HER2, human epidermal growth factor receptor 2.

In this study, the median follow‐up period was 27.2 months. The median PFS was 10.9 months (95% CI: 7.5–13.3 months) in the HER2‐low subgroup and 8.0 months (95% CI: 5.4–17.6 months) in the HER2‐zero subgroup (hazard ratio: 0.92; 95% CI: 0.64–1. 30; *p* = 0. 65) in the CDK4/6 inhibitor plus ET cohort, indicating no significant difference in the PFS of the two subgroups (Figure 1a **)**. Meanwhile, within the ET alone cohort, the HER2‐low subgroup had a significantly shorter median PFS of 5.6 months (95% CI: 3.2–8.4 months) compared to the median PFS of 17.0 months (95% CI: 11.0 months to not reached) of the HER2‐zero subgroup (hazard ratio: 2.82; 95% CI: 1.34–5.93; *p* = 0.0044) (Figure 1b). Additionally, in the ET alone cohort, the HER2‐low subgroup exhibited a slightly lower median OS compared to the HER2‐zero subgroup, although the difference was not statistically significant (36.2 vs. 42.4 months; hazard ratio: 1.41; 95% CI: 0.66–2.99; *p* = 0.36) (Figure 2). Similarly, no significant difference was observed in the median OS of the HER2‐low and HER2‐zero subgroups in the CDK4/6 inhibitor plus ET cohort (27.2 vs. 20.1 months; hazard ratio: 1.33; 95% CI: 0.50–1.09; *p* = 0.14).

Similar results were observed for the ORR (Table 3). In the ET alone cohort, the ORR was significantly lower in the HER2‐low subgroup compared to the HER2‐zero subgroup (10.5% vs. 40.0%, *p* = 0.047). Conversely, no significant difference was observed in the ORR of the HER2‐low and HER2‐zero subgroups in the CDK4/6 inhibitor plus ET cohort (15.7% vs. 11.5%; *p* = 0.398). Additionally, no significant difference was observed in the DCR of the HER2‐low and HER2‐zero subgroups in either the ET alone cohort (63.2% vs. 80.0%; *p* = 0.460) or the CDK4/6 inhibitor plus ET cohort (69.6% vs. 67.2%; *p* = 0.748) (Table 3).

**TABLE 3:** Comparison of the treatment efficacy between the CDK4/6 inhibitor plus ET cohort and ET alone cohort based on the HER2 status (*n* %).

Response rate	CDK4/6 inhibitor plus ET cohort			ET alone cohort		
	HER2‐low	HER2‐zero	*p*‐value^a^	HER2‐low	HER2‐zero	*p*‐value^a^
CR	0 (0)	0 (0)		0 (0)	1 (7.7%)	
PR	18 (15.4%)	7 (11.3%)		4 (9.8%)	3 (23.1%)	
SD	62 (53.0%)	34 (54.8%)		20 (48.8%)	4 (30.8%)	
PD	35 (29.9%)	20 (32.3%)		14 (34.1%)	2 (15.4%)	
NE	2 (1.7%)	1 (1.6%)		3 (7.3%)	3 (23.1%)	
ORR (CR + PR)	15.70%	11.50%	0.398	10.50%	40.00%	0.047*
DCR (CR + PR + SD)	69.60%	67.20%	0.748	63.20%	80.00%	0.460

Abbreviations: CDK4/6, cyclin‐dependent kinase 4/6; CR, complete response; DCR, disease control rate; ET, endocrine therapy; NE, none evaluate; ORR, objective response rate; PD, progressive disease; PR, partial response; SD, stable disease.

* *p <* 0.05.

^a^ Pearson's Chi‐squared test; Fisher's exact test.

Based on the above results, univariate and multivariate Cox regression analyses were performed for the ET alone and CDK4/6 inhibitor plus ET cohorts. The univariate analysis indicated that treatment lines and the presence of visceral metastasis were correlated with PFS (*p* < 0.05) in the CDK4/6 inhibitor plus ET cohort. Meanwhile, multivariate analysis revealed that second‐line treatment or beyond (hazard ratio: 2.0; 95% CI: 1.4–2.8; *p* = 0.00035) and the presence of visceral metastasis (hazard ratio: 1.6; 95% CI: 1.1–2.4; *p* = 0.027) were significantly associated with poorer outcomes in the CDK4/6 inhibitor plus ET cohort (Table 4). Conversely, the univariate analysis indicated that HER2 status, age, and TFI were associated with PFS (*p* < 0.05) in the ET alone cohort. Moreover, multivariate analysis confirmed that HER2‐low (hazard ratio: 2.3; 95% CI: 1.1–5; *p* = 0.029) and age ≥65 years (hazard ratio: 0.44; 95% CI: 0.22–0.88; *p* = 0.00035) were independent prognostic factors of MBC in the ET alone cohort (Table 5).

**TABLE 4:** Prognostic variables for PFS in the univariate and multivariate analyses of the CDK4/6 inhibitor plus ET cohort.

Variables	Univariate analysis		Multivariate analysis	
	HR (95% CI)	*p*‐value	HR (95% CI)	*p*‐value
HER2‐low vs. HER2‐zero	0.92 (0.64–1.3)	0.65		
ER or PR‐positive vs. ER and PR‐positive	0.94 (0.56–1.6)	0.81		
Age ≥65 years vs. <65 years	1.1 (0.65–2)	0.67		
Second‐line treatment or beyond vs. first‐line treatment	2 (1.4–3)	0.00014**	2 (1.4–2.8)	0.00035**
Fulvestrant vs. AI	1.3 (0.93–1.9)	0.12		
Visceral vs. nonvisceral metastasis	1.7 (1.1–2.6)	0.011*	1.6 (1.1–2.4)	0.027*
De novo vs. recurrent disease	0.76 (0.45–1.3)	0.3		

Abbreviations: AI, aromatase inhibitors; CDK4/6, cyclin‐dependent kinase 4/6; CI, confidence interval; ER, estrogen receptor; ET, endocrine therapy; HER2, human epidermal growth factor receptor 2; HR, hazard ratio; PFS, progression‐free survival; PR, progesterone receptor.

* *p <* 0.05.

** *p <* 0.01.

**TABLE 5:** Prognostic variables for PFS in the univariate and multivariate analyses of the ET alone cohort.

Variables	Univariate analysis		Multivariate analysis	
	HR (95% CI)	*p*‐value	HR (95% CI)	*p*‐value
HER2‐low vs. HER2‐zero	2.8 (1.3–5.9)	0.0044**	2.3 (1.1–5)	0.029*
ER or PR‐positive vs. ER and PR‐positive	0.56 (0.22–1.4)	0.23		
Age ≥ 65 years vs. < 65 years	0.37 (0.18–0.74)	0.0047**	0.44 (0.22–0.88)	0.021*
Fulvestrant vs. AI	0.75 (0.41–1.3)	0.33		
TFI ≥12 months vs. < 12 months	0.36 (0.18–0.69)	0.0024**	0.5 (0.24–1)	0.055
Visceral vs. nonvisceral metastasis	1.2 (0.63–2.1)	0.63		

Abbreviations: AI, aromatase inhibitors; CI, confidence interval; ER, estrogen receptor; ET, endocrine therapy; HER2, human epidermal growth factor receptor 2; HR, hazard ratio; PFS, progression‐free survival; PR, progesterone receptor; TFI, treatment‐free interval.

* *p <* 0.05.

** *p <* 0.01.

### Subgroup analysis

Subgroup analysis was performed according to specific baseline characteristics. In the CDK4/6 inhibitor plus ET cohort, no significant differences were observed between the HER2‐low and HER2‐zero subgroups, regardless of treatment lines (Figure S1), presence of visceral metastasis (**Figure** **S2**), or the combination of ET agents (Figure S3). However, in the ET alone cohort, the prognosis of the HER2‐low subgroup was significantly worse than that of the HER2‐zero subgroup, particularly among patients with visceral metastasis (*p* = 0.0033) (Figure S4) and patients receiving aromatase inhibitor (AI) treatment (*p* = 0.0069) (Figure S5). No significant differences were observed between the HER2‐low and HER2‐zero subgroups across different TFI categories (Figure S6).

## DISCUSSION

This is the first study to conduct a comprehensive analysis of the impact of HER2‐low expression on the efficacy of ET with or without CDK4/6 inhibitors. Our findings demonstrated that HER2‐low status only affected the efficacy of traditional ET, but not the efficacy of CDK4/6 inhibitor plus ET in advanced BC patients. Specifically, the HER2‐low and HER2‐zero patients treated with CDK4/6 inhibitor plus ET showed no significant difference in the PFS and ORR, while HER2‐low patients treated with ET alone had a significantly shorter PFS and lower ORR than HER2‐zero patients treated with ET alone. These results suggested that HER2‐low status may serve as a predictive factor for response to ET but not for CDK4/6 inhibitor plus ET.

These results are consistent with previous reports, suggesting that low HER2 expression is not a predictive biomarker for CDK4/6 inhibitor efficacy.^7^, ^9^, ^10^, ^11^, ^12^ Shao et al.^9^ analyzed data from 45 HR‐positive HER2‐negative MBC patients who received palbociclib plus AI or fulvestrant therapy and found no significant differences in the clinical outcomes, including PFS, OS, or ORR, of the HER2‐low and HER2‐zero patients. Additionally, a large‐scale study conducted at MD Anderson Cancer Center reported no association between low HER2 expression and clinical outcomes of 919 MBC patients treated with first‐line ET in combination with CDK4/6 inhibitors.^12^ Similarly, another multicenter retrospective cohort study reported no significant difference between the PFS of HER2‐low and HER2‐zero patients (*n* = 165) treated with first‐line ET plus palbociclib.^7^ However, a few studies have reported contrasting results.^6^, ^13^ A retrospective study involving 428 MBC patients showed that HER2‐low status was independently associated with worse PFS and OS compared with HER2‐zero status.^13^ This discrepancy may be attributed to differences in the study populations (such as demographic and tumor‐related characteristics), clinical treatments (including different ET agents or CDK4/6 inhibitors), and subsequent treatment strategies (particularly trastuzumab deruxtecan). Our results indicate that HER2‐low status was associated with inferior PFS and ORR in the ET alone group, which may partly account for the worse prognosis of HR‐positive HER2‐low MBC patients, compared to HER2‐zero patients.^14^ However, large‐scale prospective studies are required to further validate these results.

Several possible mechanisms may underlie the discrepancy in the efficacy of ET between HER2‐low and HER2‐zero BC patients. One potential explanation is that the crosstalk between the ER and HER2 pathways can lead to endocrine resistance.^15^ CDK4 and CDK6 act as downstream effectors of ER and HER2 pathways, and CDK4/6 inhibitors, such as palbociclib, can block HER2 signaling‐mediated cell cycle progression and proliferation.^16^, ^17^ Therefore, the efficacy of CDK4/6 inhibitors in combination therapy may be less affected by HER2 status. Another possible explanation is the differences in the genetic background between the HER2‐low and HER2‐zero tumors. HER2‐low tumors may have different molecular alterations, such as mutations in PIK3CA, PTEN loss, or activation of the MAPK pathway,^18^, ^19^ which may affect their sensitivity to ET. Notably, the exact mechanism underlying the differential treatment effect of HER2‐low expression requires further investigation. Therefore, additional molecular studies, such as transcriptomic and proteomic analysis, are needed to identify the molecular differences between HER2‐low and HER2‐zero tumors and their potential implications for treatment selection.

Despite notable differences in the PFS and ORR, no significant difference was observed in the OS of HER2‐low and HER2‐zero subgroups in either the CDK4/6 inhibitor plus ET or ET alone cohorts in this study. These results suggest that while HER2‐low expression may influence short‐term treatment response, it might not exert a substantial impact on long‐term survival outcomes. The absence of a survival difference underscores the complexity of factors contributing to OS, including unrelated causes of death, subsequent lines of effective therapy, and the emergence of resistance mechanisms. However, the findings of this study have important clinical implications, as they emphasize the need for integrating HER2 status into personalized treatment strategies in HR‐positive MBC. Considering the comparatively poorer prognosis of HER2‐low patients treated with traditional ET alone, clinicians can develop personalized treatment strategies for HER2‐low and HER2‐zero patients.

Despite several advantages, such as prospective data collection and an extended follow‐up period, this study had a few limitations. First, the sample size of the HER2‐zero subgroup in the ET alone cohort was relatively small; thus, these findings should be interpreted with caution. Furthermore, the definition of HER2‐low expression used in this analysis was not based on the latest guidelines for HER2 testing,^20^ which may affect the accuracy of the results. Therefore, further studies are needed to confirm these findings and to investigate the underlying biological mechanisms of HER2‐low status in ET response.

In conclusion, our prospective study suggests that HER2‐low expression may serve as a predictive biomarker for the efficacy of ET but not for CDK4/6 inhibitor plus ET. Overall, our study highlights the importance of considering HER2 status in the management of HR‐positive advanced BC and provides insights into the potential role of CDK4/6 inhibitors in HER2‐low BC patients. These findings may have implications in the development of personalized treatment strategies for HR‐positive MBC patients to improve their prognosis.

## AUTHOR CONTRIBUTIONS

Yun Wu: Conceptualization, data curation, formal analysis, validation, visualization, writing—original draft and writing—review and editing. Hongnan Mo: Conceptualization, data curation, formal analysis, validation and visualization. Hangcheng Xu: Conceptualization, data curation, formal analysis, validation and visualization. Yan Wang: Data curation and validation. Jiayu Wang: Conceptualization, funding acquisition, methodology, supervision and writing—review and editing. Fei Ma: Conceptualization, funding acquisition, methodology, supervision and writing—review and editing. Binghe Xu: Conceptualization, funding acquisition, methodology, supervision and writing—review and editing.

## FUNDING INFORMATION

This work was supported by the National Key Research and Development Program of China (2021YFF1201300) and the CAMS Innovation Fund for Medical Sciences (2021‐I2M‐1‐014).

## CONFLICT OF INTEREST STATEMENT

The authors have no relevant financial or nonfinancial interests to disclose.

## Supporting information


**Figure S1.** Kaplan‐Meier estimates of progression‐free survival of patients who received first‐line treatment (a) and subsequent lines of treatment (b) in the CDK4/6 inhibitors plus ET cohort stratified by HER2 status. CDK4/6, cyclin‐dependent kinase 4/6; ET, endocrine therapy; HER2, human epidermal growth factor receptor 2.
**Figure S2.** Kaplan‐Meier estimates of progression‐free survival of patients with nonvisceral metastasis (a) and visceral metastasis (b) in the CDK4/6 inhibitors plus ET cohort stratified by HER2 status. CDK4/6, cyclin‐dependent kinase 4/6; ET, endocrine therapy; HER2, human epidermal growth factor receptor 2.
**Figure S3.** Kaplan‐Meier estimates of progression‐free survival of patients who received combination agents with AI (a) and fulvestrant (b) in the CDK4/6 inhibitors plus ET cohort stratified by HER2 status. CDK4/6, cyclin‐dependent kinase 4/6; ET, endocrine therapy; HER2, human epidermal growth factor receptor 2; AI, aromatase inhibitors.
**Figure S4.** Kaplan‐Meier estimates of progression‐free survival of patients with nonvisceral metastasis (a) and visceral metastasis (b) in the ET alone cohort stratified by HER2 status. ET, endocrine therapy; HER2, human epidermal growth factor receptor 2.
**Figure S5.** Kaplan‐Meier estimates of progression‐free survival of patients who received combination agents with AI (a) and fulvestrant (b) in the ET alone cohort stratified by HER2 status. ET, endocrine therapy; HER2, human epidermal growth factor receptor 2; AI, aromatase inhibitors.
**Figure S6.** Kaplan‐Meier estimates of progression‐free survival of patients with TFI<12 month (a) and TFI ≥12 months (b) in the ET alone cohort stratified by HER2 status. ET, endocrine therapy; HER2, human epidermal growth factor receptor 2; TFIs, treatment free intervals.

## Data Availability

All data generated or analyzed during this study are included in this published article.
