# Supplementary material for: Impact of HER2‐low expression on the efficacy of endocrine therapy with or without CDK4/6 inhibitor in HR‐positive/HER2‐negative metastatic breast cancer: A prospective study
Source: Thorac Cancer. 2024 Mar 13;15(12):965–73. doi: 10.1111/1759-7714.15282 (PMC11045331; doi:10.1111/1759-7714.15282)
Supplement: Supplementary file 1 — Figure S1. Kaplan‐Meier estimates of progression‐free survival of patients who received first‐line treatment (a) and subsequent lines of treatment (b) in the CDK4/6 inhibitors plus ET cohort stratified by HER2 status. CDK4/6, cyclin‐dependent kinase 4/6; ET, endocrine therapy; HER2, human epidermal growth factor receptor 2. Figure S2. Kaplan‐Meier estimates of progression‐free survival of patients with nonvisceral metastasis (a) and visceral metastasis (b) in the CDK4/6 inhibitors plus ET cohort stratified by HER2 status. CDK4/6, cyclin‐dependent kinase 4/6; ET, endocrine therapy; HER2, human epidermal growth factor receptor 2. Figure S3. Kaplan‐Meier estimates of progression‐free survival of patients who received combination agents with AI (a) and fulvestrant (b) in the CDK4/6 inhibitors plus ET cohort stratified by HER2 status. CDK4/6, cyclin‐dependent kinase 4/6; ET, endocrine therapy; HER2, human epidermal growth factor receptor 2; AI, aromatase inhibitors. Figure S4. Kaplan‐Meier estimates of progression‐free survival of patients with nonvisceral metastasis (a) and visceral metastasis (b) in the ET alone cohort stratified by HER2 status. ET, endocrine therapy; HER2, human epidermal growth factor receptor 2. Figure S5. Kaplan‐Meier estimates of progression‐free survival of patients who received combination agents with AI (a) and fulvestrant (b) in the ET alone cohort stratified by HER2 status. ET, endocrine therapy; HER2, human epidermal growth factor receptor 2; AI, aromatase inhibitors. Figure S6. Kaplan‐Meier estimates of progression‐free survival of patients with TFI<12 month (a) and TFI ≥12 months (b) in the ET alone cohort stratified by HER2 status. ET, endocrine therapy; HER2, human epidermal growth factor receptor 2; TFIs, treatment free intervals. [file TCA-15-965-s001.docx]

**Supplementary Materials**

**Figure S1. Kaplan-Meier estimates of progression-free survival of patients who received first-line treatment (A) and subsequent lines of treatment (B) in the CDK4/6 inhibitors plus ET cohort stratified by HER2 status.** CDK4/6, cyclin-dependent kinase 4/6; ET, endocrine therapy; HER2, human epidermal growth factor receptor 2.

**
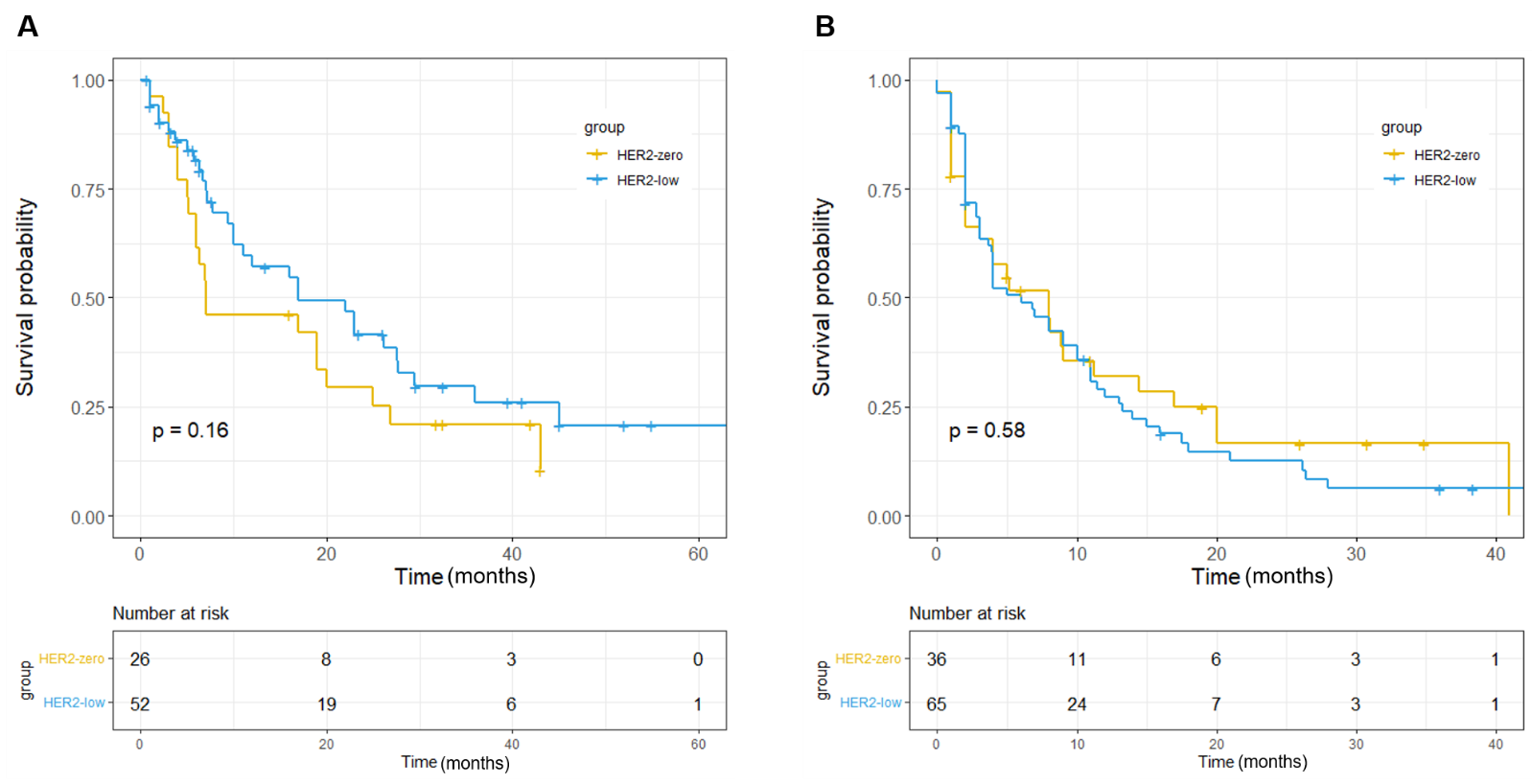
**

**Figure S2. Kaplan-Meier estimates of progression-free survival of patients with non-visceral metastasis (A) and visceral metastasis (B) in the CDK4/6 inhibitors plus ET cohort stratified by HER2 status.** CDK4/6, cyclin-dependent kinase 4/6; ET, endocrine therapy; HER2, human epidermal growth factor receptor 2.

**
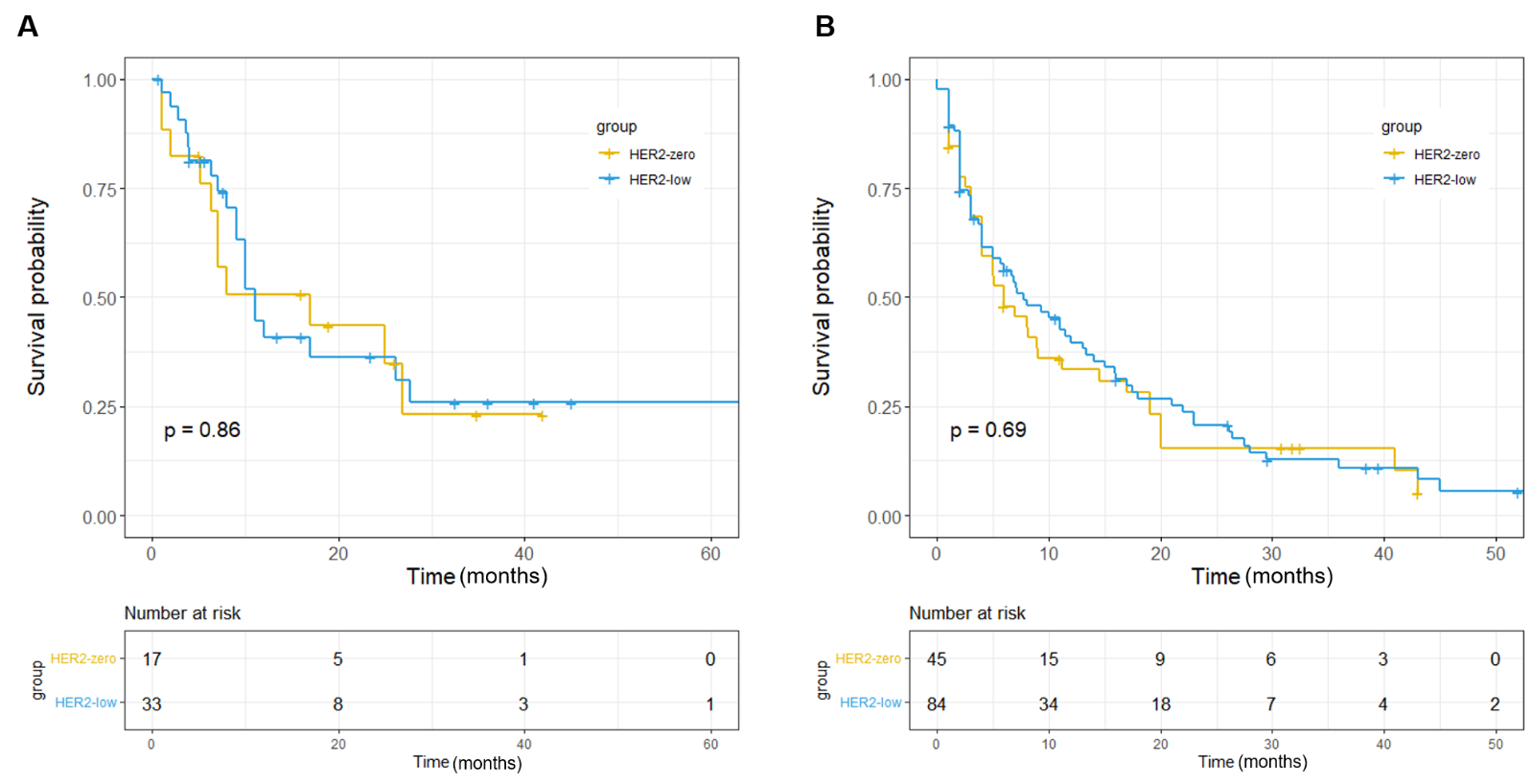
**

**Figure S3. Kaplan-Meier estimates of progression-free survival of patients who received combination agents with AI (A) and fulvestrant (B) in the CDK4/6 inhibitors plus ET cohort stratified by HER2 status.** CDK4/6, cyclin-dependent kinase 4/6; ET, endocrine therapy; HER2, human epidermal growth factor receptor 2; AI, aromatase inhibitors.

**
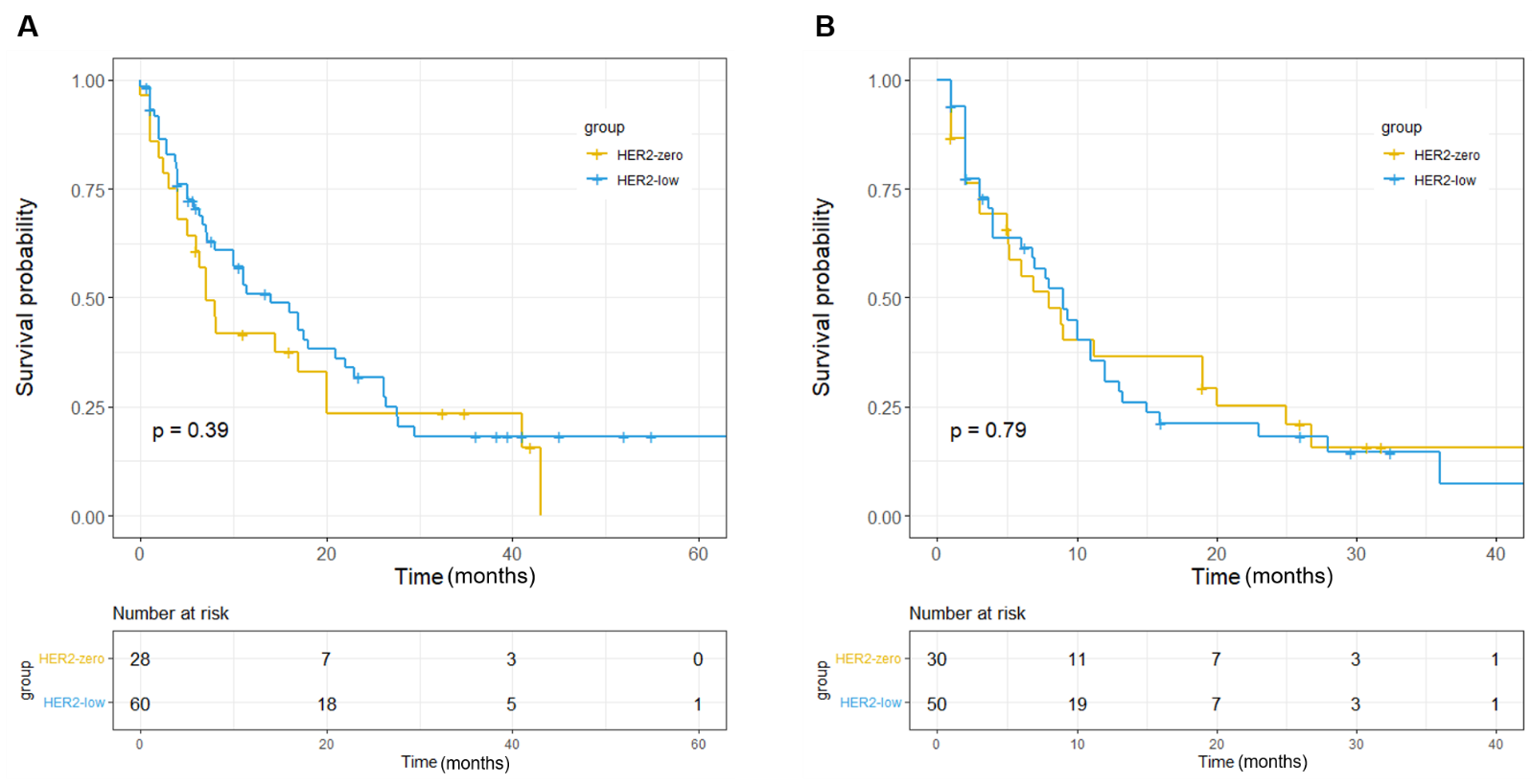
**

**Figure S4. Kaplan-Meier estimates of** **progression-free survival of patients with non-visceral metastasis (A) and visceral metastasis (B) in the ET alone cohort stratified by HER2 status.** ET, endocrine therapy; HER2, human epidermal growth factor receptor 2.

**
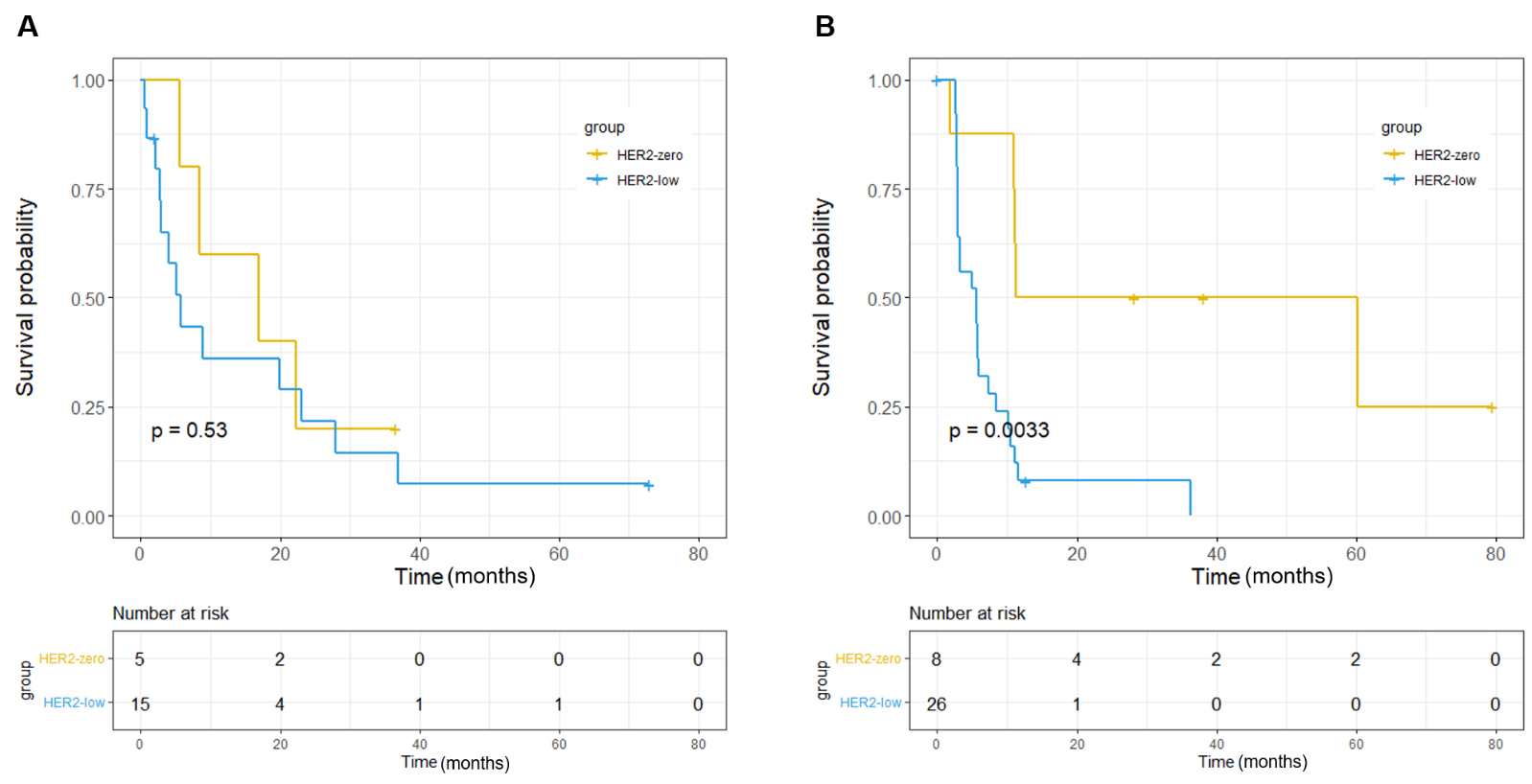
**

**Figure S5. Kaplan-Meier estimates of progression-free survival of patients who received combination agents with AI (A) and fulvestrant (B) in the ET alone cohort stratified by HER2 status.** ET, endocrine therapy; HER2, human epidermal growth factor receptor 2; AI, aromatase inhibitors.

**
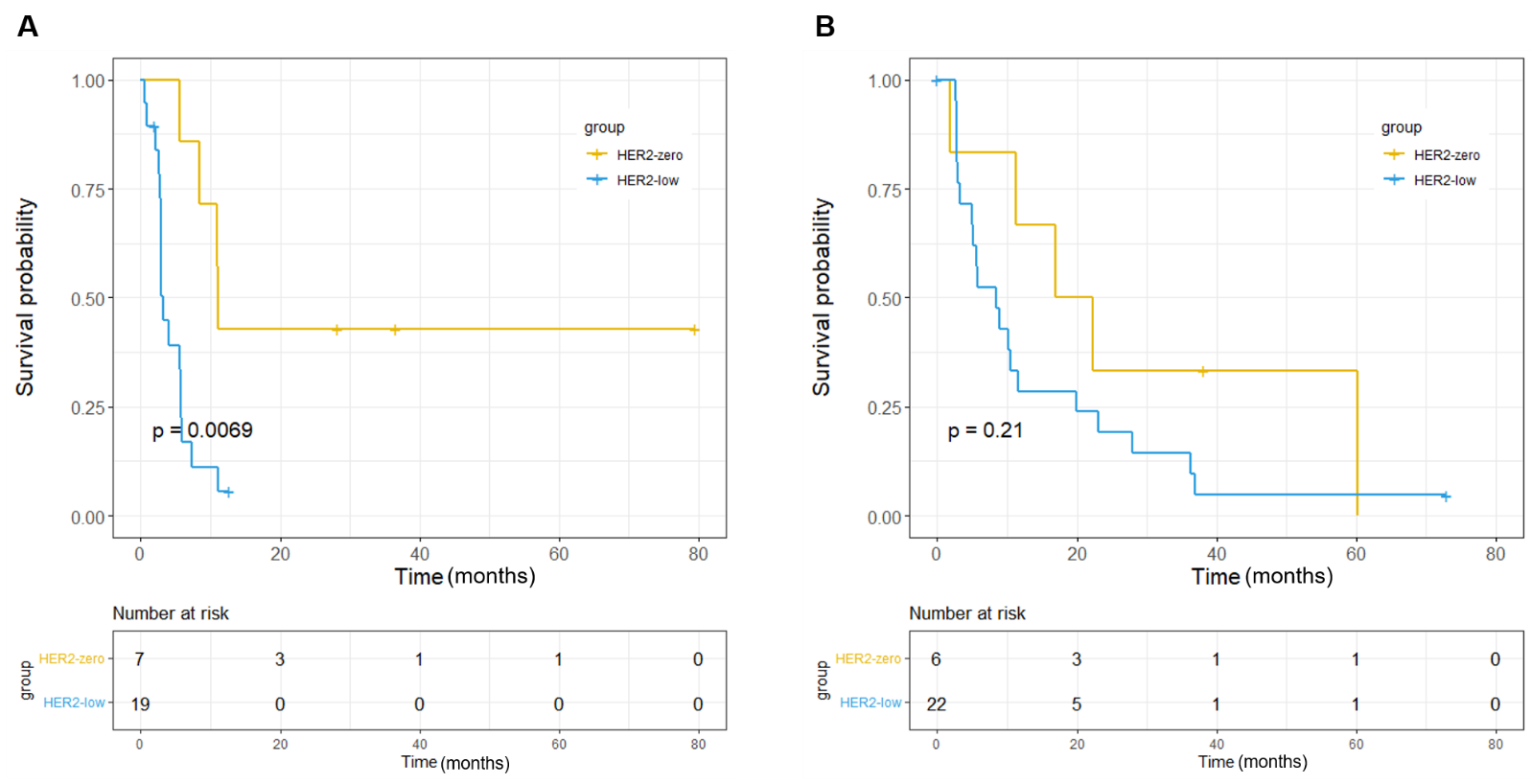
**

**Figure S6. Kaplan-Meier estimates of progression-free survival of patients with TFI＜12 month (A) and TFI ≥12 months (B) in the ET alone cohort stratified by HER2 status.** ET, endocrine therapy; HER2, human epidermal growth factor receptor 2; TFIs, treatment free intervals.

**
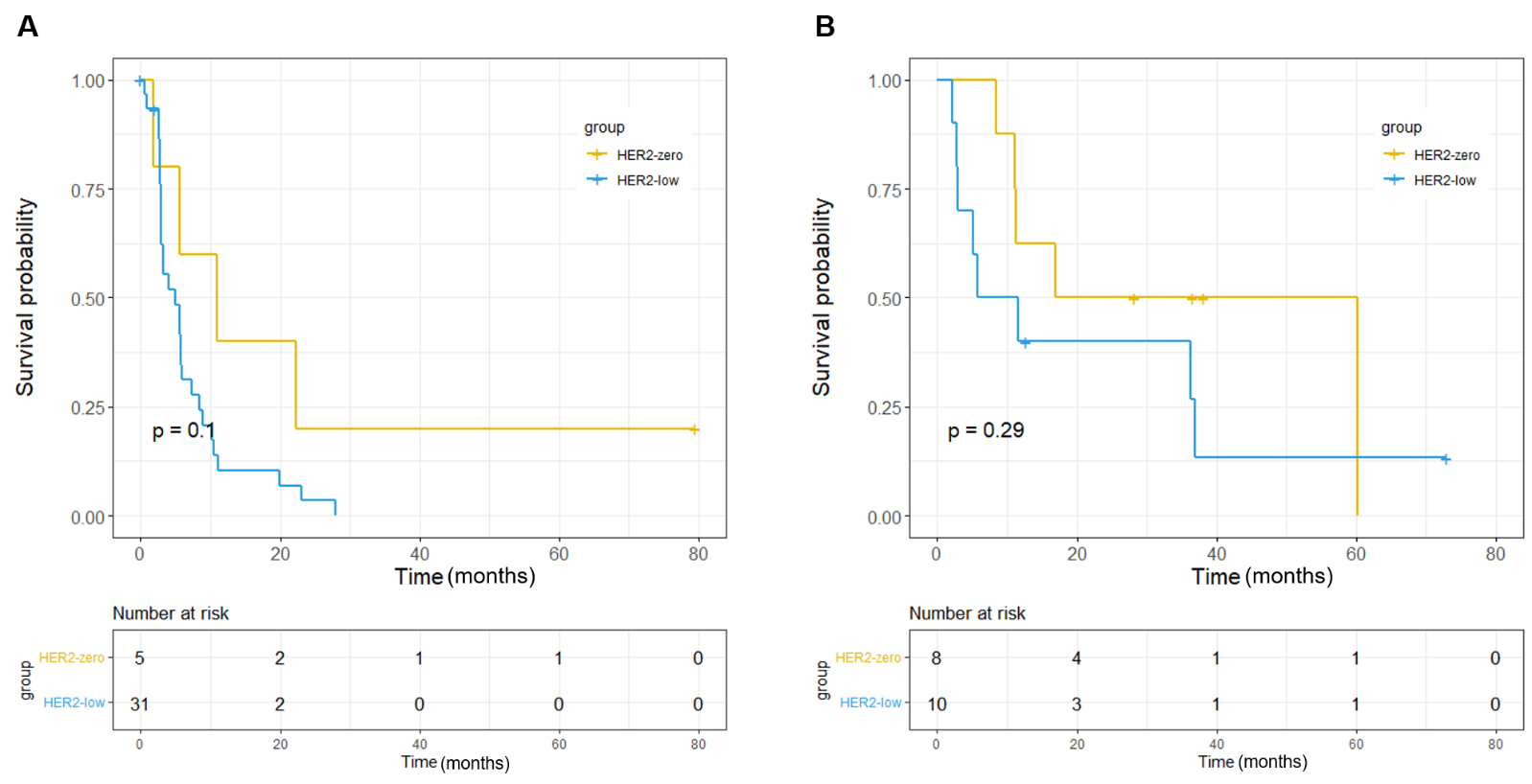
**
